# Prevalence of SARS-CoV-2 Infection in a Sample of Health Workers in Two Health Departments of the Valencian Community in Spain

**DOI:** 10.3390/ijerph19010066

**Published:** 2021-12-22

**Authors:** Kenan Rodríguez de Limia Ramírez, Nicolás Ruiz-Robledillo, José Luis Duro-Torrijos, Vicente García-Román, Natalia Albaladejo-Blázquez, Rosario Ferrer-Cascales

**Affiliations:** 1 Human Resources Service, University Hospital of Vinalopó, 03293 Elche, Spain; krodriguez@vinaloposalud.com; 2 Department of Health Psychology, University of Alicante, 03690 Alicante, Spain; natalia.albaladejo@ua.es (N.A.-B.); rosario.ferrer@ua.es (R.F.-C.); 3 Research and Teaching Service, University Hospital of Vinalopó, 03293 Elche, Spain; 4 Preventive Medicine Service, University Hospital of Vinalopó, 03293 Elche, Spain; garcia_vicrom@gva.es

**Keywords:** COVID-19, SARS-CoV-2, healthcare workers, active infection diagnostic test

## Abstract

Health care personnel constitutes the most vulnerable group of professionals, as they are employed in a work context with higher exposure to SARS-CoV-2 infection. This study aims to estimate the prevalence of SARS-CoV-2 infection in health personnel (*n* = 2858) of two health departments in the Valencian community between March 2020 and April 2021, as well as the sociodemographic and work variables predicting higher infection prevalence in this group. A cross-sectional descriptive study was performed on health workers from the health departments of Torrevieja and Elche-Crevillente of the Valencian Community (Spain). After obtaining the samples, the cases were identified through an active infection diagnostic test (AIDT). The analyzed variables were: sex, age (18–34/35–49/>50 years), professional category, health care, risk service, and AIDT. A total of 2858 staff members were studied. Of them, 55.4% (1582) underwent an AIDT, with 9.7% (277) of positive cases. Infection predominated in the age group of 18 to 34 years, 12.6% (OR = 1.98, 95% CI [1.26, 3.11]); nurses, 12.1% (OR = 1.5, 95% CI [1.00, 2.23]); and at-risk services, 11.4% (OR = 1.3, 95% CI [1.06, 1.81]). A very low positivity rate was identified in the health personnel linked to the health departments analyzed during the 14 months of the study period. Based on our results, prevention strategies could focus more intensively on the most at-risk groups, specifically young nurses who work in at-risk services, mainly in emergency and internal medicine.

## 1. Introduction

On 30 January 2020, the World Health Organization (WHO) declared the SARS-CoV-2 outbreak a public health emergency of international concern, reaching a pandemic status on March 11 [1,2]. Its rapid community transmission led to the implementation of exceptional measures by the health authorities to mitigate the consequences of infection and the mortality rates in the population and health care systems worldwide [3].

Spain was one of the first European countries affected, after Italy. Community transmission and the increase in SARS-CoV-2 cases led the Spanish government to decree the confinement of the population from 14 March until 15 July 2020 [4]. Massive quarantine measures were also implemented by countries such as India, France, Italy, New Zealand, Poland, and the United Kingdom, among others. Overall, more than half of the world’s population was under lockdown by early April 2020 [5,6].

Although the entire population is susceptible to contracting SARS-CoV-2 infection, health personnel constitutes the most vulnerable group of professionals, as they are in a work environment with higher exposure to the virus, mainly due to the health care provided to potentially contagious people in their practice, as well as the shortage of personal protective equipment (PPE), excessively long working hours due to staff shortages, prolonged exposure to a large number of infected patients, inadequate training in prevention, and the need for increased screening measures for infection control of asymptomatic SARS-CoV-2 patients—reasons that exacerbated exposure at the beginning of the pandemic and constituted a central problem [7,8,9,10]. In addition to the risks derived from the work activity of health personnel, we must add the risks to the general population, such as the potential contagion in the community’s social relationships and the coexistence with relatives at home, among others [11,12,13,14,15].

Likewise, Spain became the country with the highest number of infected health professionals in the world [15]. According to the data reported by the European Center for Disease Control and Prevention in the first months of the pandemic, in April 2020, 20% of the population affected by SARS-CoV-2 in Spain belonged to the group of health professionals, compared to 10% reported in Italy and 3.8% in China [15].

The latest report published by the “Red Nacional de Vigilancia Epidemiológica” (RENAVE, National Epidemiological Surveillance Network), corresponding to the analysis of the cases registered in health personnel from the beginning of the pandemic until 11 May 2020, indicated that, out of 250,273 confirmed cases, 16.4% (40,961) belonged to this group [16].

At present, the total number of health workers infected with SARS-CoV-2 is unknown due to the absence of epidemiological studies with global data [17]. This is because the main published research on this group of workers focuses on the initial months of the pandemic, between March and June 2020 [18,19,20,21,22,23,24]. This fact entails a significant limitation of the available results in the current literature in this regard, taking into account the importance of analyzing the evolution of the contagion in health workers over time, in order to analyze the possible effectiveness of prevention and intervention implemented strategies. For this reason, it would be interesting to have new investigations that analyze the infection data in the group of Spanish health workers during a broader chronological window, which allows obtaining a global vision of the behavior of the health context derived from the pandemic [13].

A general perspective, in contrast to the trend of the existing literature, more focused on the evaluation of the initial impact of the pandemic, which constitutes the first step to determine whether the infection prevention and control programs (IPCP) established in health centers have been effective in quantifying the impact on health personnel and analyzing the clinical and epidemiological characteristics of the affected health workers [25]. Undoubtedly, this is essential for the proper functioning of health environments and to guarantee the safety of this group of professionals. The adequate preparation and protection of health professionals will result in greater control of the pandemic, which will have an impact on the lower morbidity and mortality of the population [25]. In this regard, it is extremely urgent to obtain information about several sociodemographic, personal, and work characteristics, which could entail risk or protective factors for contagion in health staff. This information, obtained from a broad temporal spectrum, could allow researchers and clinicians to evaluate the effectiveness of the implemented prevention strategies in Spain and focus on the most at-risk groups that have shown more contagion rates. The results derived from these wider analyses could reveal very significant information to deeply evaluate the efficacy of the developed strategies, especially in a higher risk context such as Spain, as one of the countries with higher prevalences of the contagion in the group of health workers. Moreover, the evaluation of the risk profile for the contagion of Spanish health workers could add very useful information to the scientific literature, as it increases the information for the most at risk health staff, to which prevention and intervention measures should be applied the most urgently.

Taking into account that, to our knowledge, very few studies have analyzed this issue over this long a time period in Spain, the main objective of this work is to estimate the prevalence of SARS-CoV-2 in health personnel with employment links to two health departments, Torrevieja and Elche-Crevillente, in the Valencian community, in the period between March 2020 and April 2021. On the other hand, another aim of the present research is to analyze the sociodemographic and work variables that could be risk factors for contagion in the evaluated population, in order to obtain a profile of the most at-risk groups of health staff.

## 2. Materials and Methods

### 2.1. Study Design

A cross-sectional retrospective observational study was carried out to determine the prevalence of SARS-CoV-2 in all of the health professionals of the health departments of Torrevieja and Elche-Crevillente of the province of Alicante in the Valencian community, according to sociodemographic and work environment variables during the period from 1 March 2020 to 30 April 2021 (14 months). These health departments are integrated into the Spanish National Public Health System and provide health coverage to 320,000 in habitants (170,000 Torrevieja and 150,000 Elche-Crevillente). Hence, the participants of the study configure a representative sample of health workers enrolled in the national Spanish health system.

For the sample, all health personnel with a contractual relationship in both health departments were selected (*n* = 2858). Once the sample was obtained, the cases were identified using the active infection diagnostic test (PDIA), through the results located in the medical records.

### 2.2. Participants

Assuming that the number of health personnel who are part of the staff of the two health departments was stable, the reference population for the calculation of the prevalence of SARS-CoV-2 was the personnel employed as of 31 December 2020, and identified, out of the total number, those who had a job relationship during the study period (*n* = 2858).

Workers hired by external companies, such as cleaning, maintenance, or restoration personnel, were excluded from the study because they did not belong to the quota of workers dependent on the basic unit of Occupational Health. The total number of health personnel with employment links to the two health institutions were 2858 professionals. The female sex predominates in both health departments, comprising 69.9% (*n* = 1988). The mean age of the health workers was 38.9 ± 9.34 years (range 18–69 years). By age group, the category between 35 and 49 years (*n* = 1451) predominated, comprising 50.8% (*p* = 0.001) (Table 1).

The largest groups of workers were the nursing and medical groups, comprising 33.9% (*n* = 970) and 25.9% (*n* = 739), respectively. Likewise, a large number of health personnel were identified in the area of specialized care, comprising 74.2% (*n* = 2122, *p* = 0.001) (Table 1).

Based on the variables of the exposed service with direct care of patients with COVID-19 (risk vs. non-risk service), out of the total number of professionals under study, there was a predominance (53.7%, *n* = 1534) of health workers with links to risk services. We highlight the areas of primary care, with 48.0% (*n* = 736), internal medicine, 20.0% (*n* = 307), and emergency, 19.2% (*n* = 294) (Table 1).

### 2.3. Variables

#### 2.3.1. Active SARS-CoV-2 Infection

Data on the presence of active SARS-CoV-2 infection were obtained through the AIDT, the polymerase chain reaction (PCR), and the antigen detection test; both tests were contemplated in the early detection, surveillance, and control strategies of COVID-19 of the Ministry of Health [22]. AIDTs were carried out on all health personnel with contractual ties that met the criteria for possible contagion assessed by the Occupational Health Service of the two health departments, during the period between 1 March 2020 and 30 April 2021. Test results were recorded in the individual digital medical record.

#### 2.3.2. Epidemiological Variables

The variables analyzed were categorized according to sex (male/female), age (18–34/35–49/>50 years), professional category (doctors/nurses/other health personnel/non-health personnel), care area (primary/specialized), AIDT (yes/no), AIDT result (positive, negative), temporal distribution of the test and result (March–June 2020/July–December 2020/January–April 2021), exposed service with direct attention to patients with COVID-19 (risk [emergency, intensive medicine, internal medicine, primary care]/no risk [remaining services]), and the cumulative incidence rate (IR) at 14 days.

### 2.4. Procedure

Data collection was carried out retrospectively and all of the indicated variables were retrieved from medical records of the health staff. The identification variables of active infection by SARS-CoV-2 were facilitated by the Central Office of Corporate Information (OCIC) of both health departments, providing the number and result of the PDIA of the study population. These data were located in the digitized medical records of the target population on 15 May 2021. The human resources departments provided the epidemiological variables. Once the sample was obtained, the main researcher was responsible for creating a data table, guaranteeing their coding and anonimization.

All the information and data about the patients or their participation in this study were considered confidential and anonymous. For this purpose, a data collection notebook was prepared whose access and treatment were reserved to the main researcher.

### 2.5. Ethical Considerations

The study contemplates the ethical principles for medical research established in the current legislation and was evaluated and approved by the Ethics Committee of Research with Medicines of the participating centers in an ordinary session held on 28 April 2021, with the protocol code: COVIDSAN.

### 2.6. Statistical Analysis

A descriptive analysis was performed to determine the sociodemographic and professional data of all the health professionals of the health departments of Torrevieja and Elche-Crevillente of the province of Alicante, in the Valencian community

We estimated the prevalence of the proportion of participants who had a positive result in AIDT.

The prevalence of SARS-CoV-2 infection was calculated with a 95% confidence interval (CI) for the entire health personnel and each of the categories of the variables. The Chi-square test was used to study the association between the presence of antibodies and each of the variables. The odds ratio (OR) and adjusted odds ratios (ORa) were performed using a logistic regression, and its 95% CI were calculated to study the magnitude of the association. The level of statistical significance used was *p* ˂ 0.05. The statistical program SPSS, version 21.0, was used.

The cumulative 14-day incidence rate in health personnel in the two health departments per 100,000 inhabitants was calculated from the total number of workers in both departments and the confirmed cases of SARS-Cov-2 infection.

## 3. Results

### 3.1. Prevalence of SARS-CoV-2 Infection in the Target Sample

Concerning testing, 55.4% (*n* = 1582) of the health personnel in both departments underwent an AIDT during the study period. The prevalence of positive cases was 9.7% (*n* = 277) of the population during the 14 months of the study (Table 1).

This study period encompasses three epidemic blocks or waves identified by the health authorities, distributed between March–June 2020 (period 1); July–December 2020 (period 2); and January–April 2021 (period 3). Our study recorded a lower number of cases in the first period, at 0.7% (*n* = 21), although similar results were achieved in periods 2 and 3, with 4.4% (*n* = 126) and 4.8% (*n* = 136) of positive cases, respectively (Table 1).

### 3.2. AIDT vs. SARS-CoV-2 in the Target Sample

In primary care health workers, 61.0% were tested (OR = 1.48, 95% CI [1.23, 1.79], *p* < 0.001). Likewise, more tests were carried out on professionals assigned to the services with the highest risk of contagion, at 58.5% (OR = 1.13, 95% CI [1.05, 1.21], *p* < 0.001). In these services with higher exposure, there were more requests for tests in primary care, at 61.0% (OR = 1.45, 95% CI [1.21, 1.75]) and internal medicine, at 60.3% (OR = 1.38, 95% CI [1.07, 1.79], *p* < 0.001) (Table 2).

According to the professional category, a higher number of AIDTs was recorded in nurses, with 57.4% (OR = 1.29, 95% CI [1.04, 1.60]), and doctors, with 56.7% (OR = 1.25, 95% CI [0.99, 1.57]) (Table 2).

### 3.3. Confirmed Cases of SARS-CoV-2 Infection in the Target Sample

Of the health personnel with a positive diagnostic test (*n* = 277), there was hardly any difference in the sex category, at 9.8% and 9.7%, of all the men and women, respectively. Higher positivity was recorded in the age group of 18 to 34 years, at 12.6% (OR = 1.94, 95% CI [1.22, 3.07]) (Table 3).

Regarding the professional category, the nursing group predominated, showing the highest records of contagion, at 12.1% (OR = 1.34, 95% CI [0.88, 2.41]), and also the category of other health personnel, at 11.1% (OR = 1.32, 95% CI [0.83, 2.08], *p* < *0*.001) (Table 3). Although the specialized care personnel registered a lower number of AIDTs, this group had a higher positivity, at 10.1%, compared to 8.6% of primary care professionals (*p* = 0.022).

Likewise, the risk services recorded the highest rates of positive cases, at 11.4% (OR = 1.23, 95% CI [1.02, 1.79], *p* < 0.001). Among them, we highlight the workers of internal medicine, at 16.3% (OR = 1.82, 95% CI [1.22, 2.70]) and emergency, at 13.9% (OR = 1.78, 95% CI [1.11, 2.70], *p* < 0.001) (Table 3).

### 3.4. Distribution of Confirmed Cases of SARS-CoV-2 Infection in the Target Sample in Chronological Periods

The percentage of COVID-19 infection among health personnel was variable depending on the periods analyzed in the sex variable. There was little difference in period 1, with women predominating (4.7%) in period 2, and men (5.7%) in period 3 (Table 4).

Health workers linked to the Emergency Department presented higher levels of contagion in period 1, between March and June 2020 (1.7%), compared to Internal Medicine health personnel, who predominated in the rest of the periods, with 5.7% in period 2 and 6.0% in period 3 (Table 4).

The professional group of nursing and the non-health personnel obtained the highest records of positive cases in the first and last study periods, at 0.9% and 6.0%, and 0.8% and 5.3%, respectively. The other health personnel category presented the highest rates of infection during the second period, at 5.8%, followed by the nursing group, at 5.7% (Table 4).

**Figure 1:**
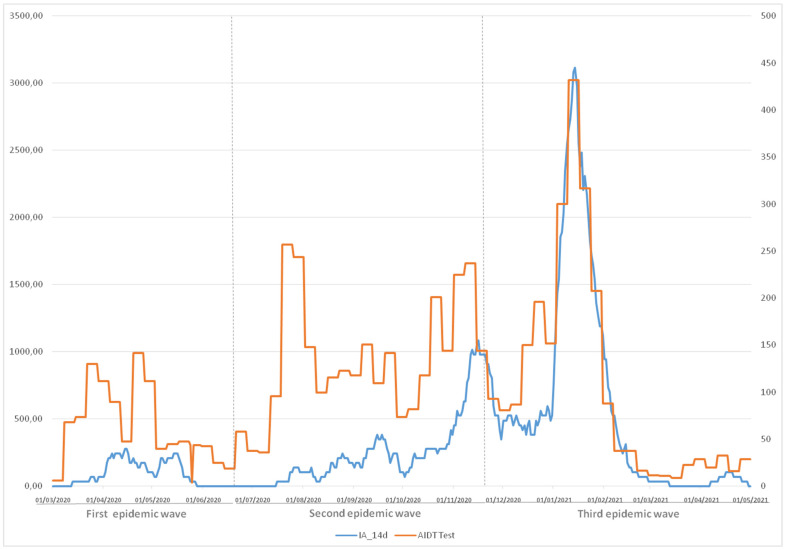
Evolution of the cumulative incidence rate (AI) and number of requests for diagnostic tests of active infection (AIDT) for SARS-CoV-2. AI 14 days/100,000 inhabitants. Number of AIDT tests.

Considering the cumulative incidence rate of reported positive cases, expressed at 14 days and per 100,000 inhabitants, the highest peak was registered in the second week of 2021, corresponding to the period between 10 and 16 January 2021, with an incidence of 3114 cases per 100,000 inhabitants (14 January 2021) in the two health departments, with a cumulative incidence similar to the number of AIDT tests requested (Figure 1).

## 4. Discussion

The present study aimed to estimate the SARS-CoV-2 infection in health personnel from two health departments, Torrevieja and Elche-Crevillente, in the Valencian community, Spain. Furthermore, this study aimed to identify the sociodemgraphic and work characteristics of health staff that could be risk factors for contagion.

Regarding the infection prevalence, the main finding of the study is that a very low positivity rate (*n* = 2858) was identified in the health personnel linked to the target health departments, at 9.7% during the 14 months of the study period. The record of positive cases was 0.7% between March and June 2020, 4.4% from July to December 2020, and 4.8% between January and April 2021, which is an increase in the number of infections similar to those reported in investigations carried out in Italy [26] and Switzerland [27]. This trend suggests that the early implementation of strict public health control measures and the demanding lockdown during the first wave were more effective in controlling the spread of SARS-CoV-2, compared to their relaxation after the summer months during the second wave [26].

These results are significantly lower than those presented in recent publications, in particular, those related to the beginning of the pandemic (Period 1), such as the studies carried out in health centers in the United States, with a higher positivity, ranging from 7.6% (Nashville, Tennessee) to 33.0% (New York) [28,29]; in the United Kingdom, with a range of 14.0% to 31.6% [18,30,31]; 21.2% in Oman [7]; 12.0% in Italy [5]; 4.1% in the Netherlands [22]; and 2.6% in Saudi Arabia [32].

Likewise, for this chronological period, several recent national publications, such as the studies carried out in health centers in Madrid, present levels of positive cases that ranged between 11.0% and 31.6% [20,23,32,33]. In Barcelona, a prevalence of 11.6% and 16.4 [14,21] was estimated. In Alicante, in the reference health department, a rate of 6.6% was reported [4].

Case investigation and the implementation of AIDTs are essential to identify the impact and development of the COVID-19 pandemic in the general population and, in particular, in health workers. In this case, the results allow the analyzing of the exposure of health workers to the virus during the health crisis, as well as evaluate the efficiency of the established safety protocols. Thus, the results presented in this research are especially relevant when analyzing the data of the infection during a broad chronological window, which includes from March 2020 to April 2021 (fourteen months), in which up to three differentiated “waves” or epidemic peaks are identified. This provides a global vision of the behavior of the pandemic in the face of the trend of the existing literature, which is more focused on the analysis of the first wave of COVID-19 [16].

These results show the importance of enhancing and updating active infection detection strategies among health personnel, in order to ensure their protection [33]. This group has proven to be the cornerstone for the control of the disease, as well as for the sustainability of the health system.

With respect to the risk factor for contagion, despite a higher rate of requests for screening in women, sex was not a significant factor, with a similar percentage of positivity in both sexes. However, when analyzing the distribution of positive cases during the three study periods, some variability was observed in their impact, which was higher in men between January and April 2021, coinciding with the existing literature [21,24,32,33,34] that states that men registered a greater impact than women. The cause could be related to the protection that women have due to X-linked immunity mechanisms, different levels of sex hormones, and levels of expression of the angiotensin II converting enzyme receptors, which is one of the gateways of the virus into the cell [35,36].

When examining the distribution by age group, our study found statistically significant differences among health personnel affected by COVID-19, with higher rates recorded in the group of people less than 34 years of age. These data are consistent with those obtained in similar studies carried out in Italy [37], China [38], and at the national level, those reported by a health department of the same province of Alicante [4]. These results suggest the possibility of associating age with the risks derived from less work experience, as well as the possibility of exposure environments other than health care [4].

The nursing professional category presented a higher prevalence of infection by SARS-CoV-2, which is consistent with previous studies conducted in Sweden [39]. These data contrast with different national and international studies, where the highest rates of contagion occurred among doctors [4,8,34]. However, when comparing the professional category during the different study periods, the rates of the personnel categorized as other health personnel and those of the non-health personnel are significant. These professional bodies, a priori, do not constitute the groups with the highest exposure to contagion and risk of transmission of the virus.

These results suggest the existence of environments of exposure to contagion other than the health centers of the two target health departments. Moreover, these data show, firstly, the effectiveness of the early implementation of the infection prevention and control programs taken in both departments, such as social distancing measures, promotion of teleworking, supervision of the adequacy of personal protective equipment, respiratory hygiene measures both for the patients treated and the health personnel, and monitoring and declaring cases to minimize the contact and exposure of health personnel. Secondly, they also show the importance of leadership of the management teams during a pandemic and the constant communication between all the agents involved (board of directors, the crisis team, senior managers, middle managers, professionals and technicians, health authorities, legal representatives of the workers, and coordination with external companies). Lastly, they stress the significance of the teamwork spirit, the continuous monitoring of the pandemic crisis, the involvement, commitment, and social discipline of the health personnel in the fulfillment of the measures adopted to avoid contagion in the work environment, and the advantages of the group synergies.

Considering the cumulative incidence rate of reported positive cases, expressed at 14 days and per 100,000 inhabitants, we observed that the study period reflects a behavior similar to that reported both nationally and regionally since the beginning of the pandemic in the RENAVE [40]. The highest peak was registered in the second week of 2021, corresponding to the period from January 10 to 16, with an incidence of 3114 cases per 100,000 inhabitants (14 January 2021) in both health departments.

This was the period with highest number of infections among the health personnel studied, which was coincident with the approval and implementation of the COVID-19 vaccine in Europe, which is the measure that has been reported as the most effective preventive factor against the illness. This preventive method was the overall result of the collective efforts of the scientific and medical communities in conjunction with the government institutions, which made it possible to develop several effective vaccines in record time [41].

At the end of 2020, the Spanish Ministry of Health approved the strategic immunization plan, which was aimed at achieving collective immunity, prioritizing vaccination among health and teaching personnel, police forces, and the elderly and vulnerable people [42]. In the case of both health departments studied, the implementation of vaccination within the preventive actions was incorporated in January and February. In the following weeks, a sharp decrease in the notifications of positive cases was observed, endorsing the effectiveness of the vaccine against COVID-19.

This represents an impact on the decrease of infections, highlighting the importance of publishing scientific studies on vaccine coverage and effectiveness; besides identifying the level of knowledge, sources of information and acceptance of the COVID-19 vaccine, as crucial instruments in order to develop effective prevention and communication strategies [43]. Hence, among the control strategies, vaccine acceptance could be one of the most effective. For that reason, information campaigns and strategies oriented to enhance vaccination, especially in health workers and undergraduate students attending healthcare courses, are extremely important, as shown by a recent study [43].

Although our study represents a significant advance in the comprehension of the COVID-19 pandemic consequences in health staff, some limitations should be considered. First, possible selection bias must be taken into account, mainly regarding the exclusion of personnel hired by external companies, whose activity is carried out in the health environment of the two health departments. Another possible limitation is the under-diagnosis of our health personnel, who had been tested with the AIDT in other health departments. We also consider the absence of identification through an analysis of the effects, symptoms, and severity of the infection in the target population as a limitation.

We note that one of the strengths of our study lies in the sample size, which included all health personnel with contractual ties from the two health departments. Another notable strength is the extensive study period analyzed. This allows us to have both a global perspective of the COVID-19 pandemic and its impact during its different periods.

## 5. Conclusions

The global impact of the COVID-19 pandemic on health personnel in the two health departments is much lower than that reported in other health institutions, both national and international. These results highlight the effectiveness of infection prevention and control programs implemented early in both departments. Likewise, the importance of preventive measures and awareness-raising actions is evident in order to avoid the contagion of the most critical professional group in the management of the pandemic.

A higher prevalence of SARS-CoV-2 infection was identified in the professional category of nursing and in the age group under 34 years. These results suggest the possibility of associating age with the risks derived from less work experience, as well as the possibility of exposure environments other than health care. For this reason, we consider it essential to strengthen the visibility of epidemiological surveillance strategies, results, data, and methods among different health professionals. 

These professionals constitute a group with a high risk of exposure to the disease during their daily practice. Their protection will help to reduce the transmission of the virus to the most vulnerable population, as well as guarantee the necessary personnel for the efficiency and operability of the health system.

## Figures and Tables

**Table 1:** Demographic characteristics of health professionals in the Elche-Crevillente and Torrevieja health departments.

	*n* = 2858	%
Age, Mean (SD) Minimum-maximum	38.99 ± 9.34 18–69	
Sex		
Male	860	30.1
Female	1988	69.9
Age group		
18–34	1000	35.0
35–49	1451	50.8
>50	407	14.3
COVID-19 exposure service		
No risk	1324	46.3
Risk	1534	53.7
Emergency	294	19.2
Intensive care	197	12.8
Internal	307	20.0
Medicine primary care	736	48.0
Professional category		
Doctors	739	25.9
Nurses	970	33.9
Other health personnel	642	22.5
Non-health staff	507	17.7
Department		
Specialized	2122	74.2
Primary	736	25.8
AIDT *		
Request	1582	55.4
No request	1276	44.6
AIDT result *		
Positive	277	9.7
Temporal distribution result		
March–June 2020	21	0.7
July–December 2020	126	4.4
January–April 2021	139	4.8

(*) AIDT. Active infection diagnostic test.

**Table 2:** Requests for the diagnostic test of active infection (AIDT) for SARS-CoV-2 in the health professionals of the Elche-Crevillente and Torrevieja health departments.

	Evaluated (*n*) 1582/2858	% 55.4	ORc 95% CI	ORa 95% CI	*p* * Value
Sex					
Male (*n* = 860)	453	52.7	Reference	Reference	0.059
Female (*n* = 1988)	1129	56.8	1.16 [0.99–1.37]	1.15 [0.97–1.36]	
Age group					0.389
18–34 (*n* = 1000)	563	56.3	1.21 [0.95–1.54]	1.17 [0.93–1.47]	
35–49 (*n* = 1451)	806	55.5	1.18 [0.94–1.47]	1.13 [0.93–1.14]	
>50 (*n* = 407)	213	52.3	Reference	Reference	
COVID-19 exposure service					0.001
No risk (*n* = 1324)	685	51.7	Reference	Reference	
Risk (*n* = 1534)	897	58.5	1.27 [0.99–1.65]	1.13 [1.05–1.21]	
Emergency (*n* = 294)	170	57.8	1.30 [1.01–1.69]	1.28 [0.99–1.65]	0.001
Intensive medicine (*n* = 197)	93	47.2	0.83 [0.61–1.12]	0.81 [0.59–1.09]	
Internal medicine (*n* = 307)	185	60.3	1.41 [1.09–1.82]	1.38 [1.07–1.79]	
Primary care (*n* = 736)	449	61.0	1.46 [1.21–1.76]	1.45 [1.21–1.75]	
Professional category					
Doctors (*n* = 739)	419	56.7	1.24 [0.98–1.55]	1.25 [0.99–1.57]	0.088
Nurses (*n* = 970)	557	57.4	1.32 [1.06–1.64]	1.29 [1.04–1.60]	
Other health personnel (*n* = 642)	347	54.0	1.19 [0.94–1.52]	1.12 [0.89–1.52]	
Non-health personnel (*n* = 507)	259	51.1	Reference	Reference	
Department					
Specialized (*n* = 2122)	1133	53.4	Reference	Reference	0.001
Primary (*n* = 736)	449	61.0	1.36 [1.15–1.62]	1.48 [1.23–1.79]	

(*) *p*-value for Chi-square test. *p* < 0.05, cut-off point of statistical significance. ORc, crude odds ratios; CI confidence interval. ORa, adjusted odds ratios for all other variables in the table.

**Table 3:** Confirmed cases of SARS-CoV-2 infection in the health professionals of the Elche-Crevillente and Torrevieja health departments.

	Positive Cases (*n*) 277/2858	% 9.7	ORc 95% CI	ORa 95% CI	*p* * Value
Sex					0.269
Male (*n* = 860)	84	9.8	Reference	Reference	
Female (*n* = 1988)	193	9.7	0.90 [0.68–1.20]	0.89 [0.67–1.18]	
Age group					
18–34 (*n* = 1000)	126	12.6	1.98 [1.26–3.11]	1.94 [1.22–3.07]	0.001
35–49 (*n* = 1451)	124	8.5	1.25 [0.80–1.95]	1.22 [0.77–1.91]	
>50 (*n* = 407)	27	6.6	Reference	Reference	
COVID-19 exposure service					0.010
No risk (*n* = 1324)	102	7.7	Reference	Reference	
Risk (*n* = 1534)	175	11.4	1.38 [1.06–1.81]	1.23 [1.02–1.79]	
Emergency (*n* = 294)	41	13.9	1.81 [1.20–2.73]	1.78 [1.11–2.70]	0.001
Intensive medicine (*n* = 197)	21	10.7	1.66 [0.98–2.83]	1.52 [0.82–2.41]	
Internal medicine (*n* = 307)	50	16.3	2.11 [1.43–3.11]	1.82 [1.22–2.70]	
Primary care (*n* = 736)	63	8.6	0.93 [0.66–1.31]	0.85 [0.58–1.23]	
Professional category					0.001
Doctors (*n* = 739)	50	6.8	0.76 [0.48–1.20]	0.77 [0.48–1.22]	
Nurses (*n* = 970)	117	12.1	1.50 [1.00–2.23]	1.34 [0.88–2.03]	
Other health personnel (*n* = 642)	71	11.1	1.45 [0.94–2.22]	1.32 [0.83–2.08]	
Non-health personnel (*n* = 507)	39	7.7	Reference	Reference	
Department					0.021
Specialized (*n* = 2122)	214	10.1	Reference	Reference	
Primary (*n* = 736)	63	8.6	0.70 [0.51–095]	0.69 [0.51–0.94]	

(*) *p*-value for Chi-square test. *p* < 0.05, cut-off point of statistical significance. ORc, crude odds ratios; CI confidence interval. ORa, adjusted odds ratios for all other variables in the table.

**Table 4:** Distribution of confirmed cases of SARS-CoV-2 infection in the healthcare professionals, according to epidemic waves.

	March–June 2020			July–December 2020			January–April 2021		
	Positive *n* = 21/2858	% (0.7)	*p* * Value	Positive *n* = 126/2858	% (4.4)	*p* * Value	Positive *n* = 139/2858	% (4.8)	*p* * Value
Sex									0.172
Male (*n* = 860)	7	0.8	0.672	32	3.7	0.665	49	5.7	
Female (*n* = 1988)	14	0.7		94	4.7		90	4.5	
Age group									0.296
18–34 (*n* = 1000)	10	1.0	0.016	64	6.4	0.005	57	5.7	
35–49 (*n* = 1451)	8	0.6		50	3.4		69	4.8	
>50 (*n* = 407)	3	0.7		12	2.9		13	3.2	
COVID-19 exposure service									0.921
No risk (*n* = 1324)	9	0.7	0.978	35	2.6	0.001	59	4.5	
Risk (*n* = 1534)	12	0.8		91	5.9		80	5.2	
Emergency (*n* = 294)	5	1.7	0.675	20	6.8	0.001	19	6.5	0.110
Intensive medicine (*n* = 197)	-	-		8	4.1		13	6.6	
Internal medicine (*n* = 307)	1	0.3		30	9.8		22	7.2	
Primary care (*n* = 736)	6	0.8		33	4.5		26	3.5	
Professional category									0.096
Doctors (*n* = 739)	5	0.7	0.078	25	3.4	0.001	22	3.0	
Nurses (*n* = 970)	9	0.9		55	5.7		58	6.0	
Other health personnel (*n* = 642)	3	0.5		37	5.8		32	5.0	
Non-health personnel (*n* = 507)	4	0.8		9	1.8		27	5.3	
Department									0.029
Specialized (*n* = 2122)	15	0.7	0.990	93	4.4	0.001	113	5.3	
Primary (*n* = 736)	6	0.8		33	4.5		26	3.5	

(*) *p*-value for Chi-square test. *p* < 0.05, cut-off point of statistical significance.

## Data Availability

Informed consent was obtained from all subjects involved in the study. Written informed consent has been obtained from the participant(s) in order to publish this paper.
